# Comprehensive Analysis of a Cancer-Immunity Cycle–Based Signature for Predicting Prognosis and Immunotherapy Response in Patients With Colorectal Cancer

**DOI:** 10.3389/fimmu.2022.892512

**Published:** 2022-05-31

**Authors:** Yufang Hou, Rixin Zhang, Jinbao Zong, Weiqi Wang, Mingxuan Zhou, Zheng Yan, Tiegang Li, Wenqiang Gan, Silin Lv, Zifan Zeng, Min Yang

**Affiliations:** ^1^ State Key Laboratory of Bioactive Substances and Function of Natural Medicine, Institute of Materia Medica, Chinese Academy of Medical Sciences and Peking Union Medical College, Beijing, China; ^2^ Clinical Laboratory, The Affiliated Hospital of Qingdao University, Qingdao, China; ^3^ Qingdao Hospital of Traditional Chinese Medicine, The Affiliated Qingdao Hiser Hospital of Qingdao University, Qingdao, China

**Keywords:** colorectal cancer, gene signature, cancer immunity, tumor microenvironment, prognosis, immunotherapy

## Abstract

Immune checkpoint blockade (ICB) has been recognized as a promising immunotherapy for colorectal cancer (CRC); however, most patients have little or no clinical benefit. This study aimed to develop a novel cancer-immunity cycle–based signature to stratify prognosis of patients with CRC and predict efficacy of immunotherapy. CRC samples from The Cancer Genome Atlas (TCGA) were used as the training set, while the RNA data from Gene Expression Omnibus (GEO) data sets and real-time quantitative PCR (RT-qPCR) data from paired frozen tissues were used for validation. We built a least absolute shrinkage and selection operator (LASSO)-Cox regression model of the cancer-immunity cycle–related gene signature in CRC. Patients who scored low on the risk scale had a better prognosis than those who scored high. Notably, the signature was an independent prognostic factor in multivariate analyses, and to improve prognostic classification and forecast accuracy for individual patients, a scoring nomogram was created. The comprehensive results revealed that the low-risk patients exhibited a higher degree of immune infiltration, a higher immunoreactivity phenotype, stronger expression of immune checkpoint–associated genes, and a superior response to ICB therapy. Furthermore, the risk model was closely related to the response to multiple chemotherapeutic drugs. Overall, we developed a reliable cancer-immunity cycle–based risk model to predict the prognosis, the molecular and immune status, and the immune benefit from ICB therapy, which may contribute greatly to accurate stratification and precise immunotherapy for patients with CRC.

## Introduction

The introduction of immunotherapy, especially immune checkpoint inhibitors (ICIs), which boost the antitumor activity of T cells and rescue immune surveillance by blocking programmed cell death protein 1 (PD-1), programmed cell death 1 ligand 1 (PD-L1), and cytotoxic T lymphocyte associated antigen 4 (CTLA-4), has elicited tremendous excitement owing to its success in various types of cancers, including melanoma (1), hepatocellular carcinoma (2), and lung cancer (3). Two PD-1–blocking antibodies, pembrolizumab and nivolumab, are major therapeutic options for mismatch repair-deficient and microsatellite instability-high (dMMR/MSI-H) tumors in patients with metastatic colorectal cancer (mCRC) (4, 5). Unfortunately, the great majority of individuals with mCRC (> 95%) have cancers that are not dMMR/MSI-H. ICIs-based immunotherapies currently offer little to no clinical benefit (6, 7). Therefore, it is crucial to establish reliable predictive biomarkers to identify subgroups that may have benefits from ICIs.

The cancer-immunity cycle is characterized by an arrangement of stepwise events required for the immune system to effectively control cancer development, integrating seven key anticancer immune steps (8). Several elements, both stimulatory and inhibitory in nature, must be coordinated at each phase of the cancer-immunity cycle (8–10). Based on the theory of the cancer-immunity cycle, cancer immunotherapy is mainly divided into two categories as described below. One class aims to boost the stimulatory immune factors, which may improve anticancer immune responses and enhance the cycle’s final self-propagation (11–13). The other class, including the PD-1/PD-L1 blockade, is intended to prevent immune effector inhibition based on immune evasion mechanisms (11–14). There is increasing evidence that the efficacy of ICIs stemmed from a thorough understanding of the dynamics of antitumor immune responses and immunosuppressive circumstances in the tumor microenvironment (TME) (15). Therefore, the foundation for adopting treatment strategies specific to each patient, which is required to facilitate the development of more effective immunotherapies, is a comprehensive evaluation of the tumor immunophenotype, including the status of the cancer-immunity cycle and immune cell infiltration of individuals. At present, ICIs are standard-of-care options for first-line or later-line treatment in patients with dMMR/MSI-H mCRC, where they show a striking antitumor activity (5–7). More importantly, several ongoing clinical trials have revealed that ICIs in conjunction with molecular-targeted therapies or radiation might offer clinical utility of ICIs with immunomodulatory effects for patients with mismatched repair-proficient and microsatellite-stable (pMMR/MSS) tumors (16). Although these findings have emphasized the necessity of ICIs for CRC, the immunological landscape and molecular properties of CRC immunophenotype, as well as their potential implications for the immunotherapy response, remain unclear.

In this study, by combining the multigene expression data sets, we developed and validated a novel cancer-immunity cycle–based signature for risk stratification, subgroup categorization, and prognosis evaluation in CRC patients. Moreover, we comprehensively investigated the signature’s link to the landscape of immune-related characteristics, immunotherapy responses, and chemotherapy sensitivity in patients with CRC. Our results revealed that this cancer-immunity cycle–based signature may be used as a potential biomarker to predict clinical prognosis and immunotherapy efficacy among patients with CRC.

## Materials and Methods

### Data Collection

Transcriptomic information and clinical data of colon adenocarcinoma (COAD) and rectum adenocarcinoma (READ) were acquired from The Cancer Genome Atlas (TCGA) database (https://portal.gdc.cancer.gov), and the entire TCGA cohort was COAD database combined with READ database. The training set consisted of data from 597 TCGA-CRC patients with accessible clinicopathological characteristics and complete follow-up information. External validation sets contained Affymetrix microarray data for colon cancer cohorts GSE39582 (n = 562) (17) and GSE37892 (n = 130) (18), which were retrieved from the Gene Expression Omnibus (GEO) database (https://www.ncbi.nlm.nih.gov/geo). The expression and localization of the corresponding proteins from CRC tumor tissues and normal tissues were assayed using immunohistochemical data based on the Human Protein Atlas (HPA) database (http://www.proteinatlas.org). Consensus molecular subtypes (CMSs) for patients in the three cohorts were downloaded from Colorectal Cancer Subtyping Consortium Synapse (19). Transcriptome RNA sequencing data for the immunotherapeutic cohort IMvigor210 (20) were downloaded from the Creative Commons 3.0 license (http://research-pub.gene.com/IMvigor210CoreBiologies).

The Tumor Immunophenotype (TIP) database (21) (http://biocc.hrbmu.edu.cn/TIP) is a web-based tool that can assess the immune microenvironment on the basis of the cancer-immunity cycle (8). From the TIP, we collected 178 signature genes engaged in the seven stages of the cancer-immunity cycle, including checkpoints, cytotoxic factors, chemokines, and major histocompatibility complex (MHC) molecules. A total of 174 signature genes obtained in the expression profile of TCGA cohort were used as candidate genes in this study.

### Signature Construction

Based on the 174 candidate genes, least absolute shrinkage and selection operator (LASSO)-Cox regression was performed using the “glmnet” R package to reduce the likelihood of overfitting. At the penalty parameter (λ_min_) = 0.032, the optimal risk model was constructed based on 13 cancer-immunity cycle–related genes, including five chemokine and chemokine receptor family members [C-C motif chemokine ligand (CCL)11, CCL19, CCL22, CCL28, C-X-C motif chemokine receptor 5 (CXCR5)], three immune checkpoint genes [indoleamine 2,3-dioxygenase 1 (IDO1), lymphocyte activating 3 (LAG3), T cell immunoglobulin and mucin domain containing 4 (TIM4)], three heat shock protein 70 (HSP70) family members [HSP70 member 1A (HSPA1A), HSP70 member 8 (HSPA8), HSP70 member 9 (HSPA9)], and two cytokines [nitric oxide synthase 2 (NOS2) and transforming growth factor beta 1 (TGFβ1)]. The risk score for each patient was calculated according to the following formula:


$$\text{Risk score}=\sum_{i=1}^{n}\text{ Coefficient }({\text{mRNA}}_{i})\times \text{Expression }({\text{mRNA}}_{i})$$

Based on the median value of the risk score, patients were divided into two groups, including the low-risk group and the high-risk group. Associations between the risk score and clinicopathological features (age, gender, race, histological type, TNM stage, clinical stage, CMS subtype, and MSI status) were analyzed, and Sankey diagrams were obtained to evaluate the correlation between the risk score and different survival outcome, clinical stage, and CMS subtype.

### Prognostic Values of the Risk Signature

The prognostic value of the cancer-immunity cycle based-signature was evaluated in TCGA data set and validated in two independent GEO data sets (GSE39582 and GSE37892). The KM curves were plotted to compare the overall survival (OS) or progression-free survival (PFS) between the low- and high-risk groups *via* R package “survival”. The result of univariate and multivariate Cox analysis was visualized as a forest plot. Additionally, the time-dependent receiver operating characteristic (ROC) curve analysis (including one-, three-, and five-year survival) was developed to indicate the specificity and sensitivity of the risk signature utilizing R package “survivalROC”. The area under the curve (AUC) value was calculated and used to designate the ROC effect.

### Construction and Validation of the Nomogram Model

The “rms” program was used to create a predicted nomogram based on independent prognostic criteria utilizing the clinical features and risk score. In the nomogram scoring system, each variable was assigned a score, and the total score was calculated by adding the scores from all factors in each sample. To determine the consistency of the nomogram prediction and clinical observation in three- and five-year OS, PFS, or relapse-free survival (RFS), calibration curves were utilized. The nomogram was evaluated using ROC curves for three- and five-year survival. Meanwhile, the concordance index (C-index) was computed to determine the nomogram’s predictive potential.

### Biological Process and Pathway Enrichment Analysis

The R package “DESeq2” was used to filter differentially expressed genes (DEGs) between high- and low-risk patients. Genes with adjusted *P* value < 0.05 and |logFC| ≥ 0.5 were considered statistically significant. Kyoto Encyclopedia of Genes and Genomes (KEGG) pathway and Gene Ontology (GO) analyses were performed for genes enriched in the low- and high-risk categories using the R package “clusterProfiler”.

Gene Set Enrichment Analysis (GSEA) was performed to distinguish hallmark pathways involved in the gene signature. The Java GSEA software (version 4.1.0) was used and “h.all.v7.2.symbols.gmt” was set as the reference database. Pathways with normalized *P* value < 0.05 and false-discovery rate (FDR) q value < 0.25 were defined as statistically significant. Top enriched pathways were selected by ranking of normalized enrichment scores (NESs).

### Assessment of Immune Cell Infiltration

We used the currently accepted methodologies to compute the immune infiltration status among samples from TCGA database to analyze the association between infiltrating immune cells and the risk signature. First, the Estimation of STromal and Immune cells in Malignant Tumor tissues using Expression data (ESTIMATE) algorithm (22) was applied to calculate StromalScore and ImmuneScore of each sample in CRC in terms of respective gene expression profiles of stromal and immune cells *via* the R package “estimate”. Then, the distribution and ratio of different kinds of infiltrating immune cells were calculated using Estimating the Proportions of Immune and Cancer cells (EPIC) (23) algorithm and Microenvironment Cell Populations-counter (MCP-counter) (24). To determine the proportions of immune cells in each risk group, a set of metagenes, containing nonoverlapping sets of genes that are representative of 28 specific immune cell subpopulations, was obtained (25). The 28 types of immune cells were quantified using a single-sample GSEA (ssGSEA) algorithm in the light of the transcriptome data and related gene sets with the R package “GSVA”. Wilcoxon rank-sum test was used to compare the content of infiltrating immune cells in CRC between the low- and high-risk groups. Furthermore, Spearman correlation analysis was applied to analyze the association between the risk score values and critical components of tumor immunity, such as effector cells, suppressor cells, immunoregulatory factors, and MHC molecules.

### Analysis of Immunotherapy Efficacy

To explore the somatic mutations in CRC between low- and high-risk patients, the Mutation Annotation Format (MAF) for TCGA-COAD and TCGA-READ cohorts was obtained from TCGA data portal (https://portal.gdc.cancer.gov) and analyzed with R package “maftools.” The number of somatic mutations and neoantigens for TCGA database was retrieved from The Cancer Immunome Atlas (TCIA) (https://tcia.at) (25). Tumor Immune Dysfunction and Exclusion (TIDE) is a machine learning approach that simulates two main processes of tumor immune escape, and may be employed to predict cancer patients’ responsiveness to ICIs (26). The TIDE score is superior to recognized immunotherapy biomarkers [tumor mutation burden (TMB), PD-L1 level, and interferon γ] for measuring the effect of anti-PD1 and anti-CTLA4 treatment (26). The TIDE score, T cell dysfunction score, and MSI score were retrieved from the TIDE portal (http://tide.dfci.harvard.edu) on the basis of normalized transcriptome data from the TCGA-CRC data set. Furthermore, IMvigor210 cohort was used as an independent validation cohort, which profiled patients with advanced urothelial cancer who received atezolizumab, an anti-PD-L1 antibody, as a treatment (20). We used the IMvigor210CoreBiologies package to extract the complete expression data and detailed clinical information. Afterward, the risk score was computed for each patient in IMvigor210 cohort, and the correlation between the risk score and response to anti-PD-L1 treatment was evaluated. Moreover, the relationships between the risk scores and the expression levels of immune checkpoint-related genes as well as distinct tumor immune phenotypes were analyzed.

### Real-Time Quantitative PCR Validation

A total of 45 CRC and matched adjacent normal (distance to cancer greater than 5 cm) tissue samples used for RT-qPCR assay were obtained from patients who had been diagnosed with CRC by pathological examination of tissue biopsy and undergone operations at The Affiliated Hospital of Qingdao University. The inclusion criteria were as follows (1): diagnosis of CRC based on pathological analysis and imaging; (2) radical resection; (3) intact data on clinicopathological findings such as gender, age, tumor size, differentiation, tumor site, histological stage, and TNM classification; (4) TNM classification according to the 8th edition of the American Joint Committee on Cancer; (5) no history of other malignancies. The following patients were excluded: (1) patients who had recurrent CRC and/or nonprimary cancers; (2) patients who received neoadjuvant chemotherapy and/or radiotherapy before operation. Informed consents were obtained from all of the participating patients. This work was approved by the Research Ethics Committee of The Affiliated Hospital of Qingdao University and was carried out in accordance with the 1964 Helsinki Declaration and its later revisions. The enrolled patients and their clinical characteristics are listed in **Supplementary Table S1**.

After washing with cold phosphate-buffered saline, tissue specimens were immediately immersed into liquid nitrogen and then transferred and stored at –80°C. Total RNA was extracted from the tissue specimens using the RNeasy kit (Beyotime, Shanghai, China, R0027). SuperScript II reverse transcriptase was used to synthesize first-strand cDNA from an RNA template (1 mg) (TaKaRa, Japan, RR047). The SYBR Green Mix (TaKaRa, Japan, RR820) was then used by the ABI 7900 HT Real-Time PCR machine to perform RT-qPCR (Applied Biosystems, California, USA). Normalization to glyceraldehyde phosphate dehydrogenase (GAPDH) yielded relative expression levels. **Supplementary Table S2** shows the sequences of the primers utilized in our study. Each patient’s risk score was determined, and the median risk score was used to sort the patients into two categories.

### Analysis of Drug Sensitivity

The pharmacogenomics database Genomics of Drug Sensitivity in Cancer (GDSC; https://www.cancerrxgene.org) was utilized to assess CRC patients’ response to chemotherapy treatments (27). The R program “oncoPredict” was used to calculate the half-maximal inhibitory concentration (IC50) (28). We selected some of the drugs commonly used for chemotherapy and molecular-targeted therapy in CRC, and analyzed the differences in sensitivity to the above drugs between the low- and high-risk categories using the Wilcoxon rank-sum test.

### Statistical Analysis

R software (version 4.1.3) with necessary packages, as well as SPSS22.0 (IBM Corp., Armonk, New York, United States) and GraphPad Prism 8.0 (GraphPad Software Inc., San Diego, CA, United States), were used for data analysis and visualization. The Wilcoxon rank-sum test or Kruskal–Wallis test was performed to analyze continuous variables; the Fisher’s exact test or chi-square test (χ2) was used to analyze categorical variables. Relationships between risk scores and expression levels of different genes were examined by Spearman’s correlation analysis. Statistical significance was defined as a *P* value lower than 0.05.

## Results

### Construction of the Cancer-Immunity Cycle–Based Signature for Patients With CRC

**Figure 1:**
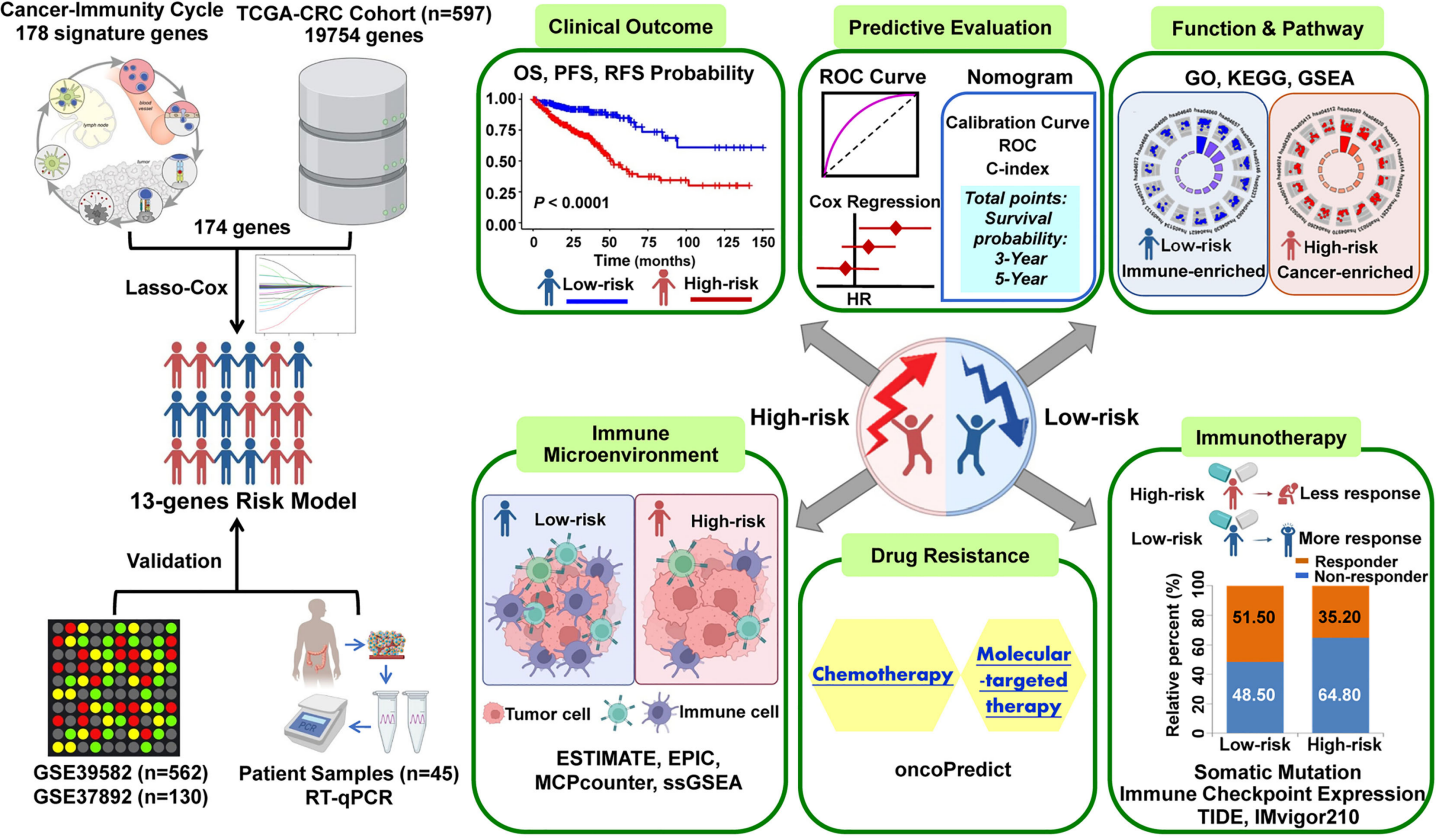
The workflow of identification of the cancer-immunity cycle–based signature for patients with CRC.

**Figure 2:**
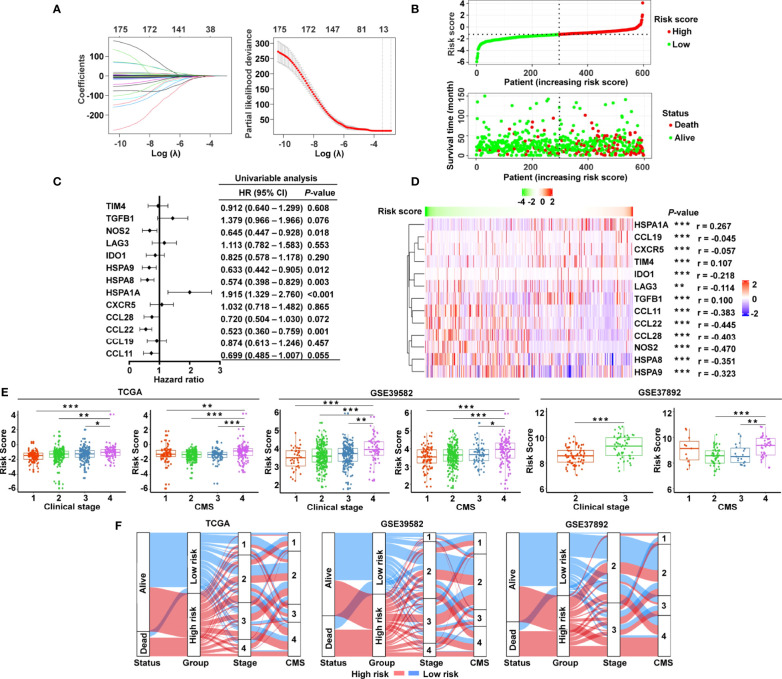
Construction of the cancer-immunity cycle–based signature for CRC patients. **(A)** Analysis of LASSO regression in TCGA database. The determination of “lambda” for optimal selection of gene signature. **(B)** The risk score distribution and patient survival status are depicted in ranked dot and scatter plots. **(C)** Forest plot of the prognostic ability of the 13 cancer-immunity cycle–related genes included in the risk signature. **(D)** Heat map of the correlations between the risk score and 13 cancer-immunity cycle–related genes constructing the risk signature. **(E)** Association between the risk score and clinicopathological features, including clinical stage and CMS in TCGA, GSE39582, and GSE37892. **(F)** Sankey diagram illustrating the flow from the two risk subgroups to different clinical outcome, clinical stage, and CMS subtype; the breadth of the flow rate is proportional to the number of patients. ***P < 0.001; **P < 0.01; *P < 0.05.

The graphic flowchart summarizes the main design of the present study (**Figure 1**). LASSO and multivariate Cox analyses for 174 cancer-immunity cycle–related genes in TCGA cohort were performed to build the optimum risk signature for evaluating the prognosis of patients with CRC. The resultant change trajectory of each independent variable is depicted in **Figure 2A** (left), followed by the LASSO regression analysis. The confidence interval under each lambda is exhibited in **Figure 2A** (right). There were 13 cancer-immunity cycle–associated genes with an optimal λ value, including five chemokine and chemokine receptor family members (CCL11, CCL19, CCL22, CCL28, CXCR5), three immune checkpoint genes (IDO1, LAG3, TIM4), three HSP70 family members (HSPA1A, HSPA8, HSPA9), and two cytokines (NOS2 and TGFβ1). To determine the clinical relevance of the candidate genes’ expression, the HPA database was used to explore the expression of the proteins encoded by these markers in CRC tumor tissues and normal tissues. Consistent with our results in TCGA (**Supplementary Figure S1A**), HSPA9 and HSPA8 were significantly upregulated in CRC tissues compared with normal tissues, and mainly localized to the cell membrane and cytoplasm in the tumor cells (**Supplementary Figure S1B**). In contrast, the protein expression levels of CCL28, HSPA1A, CXCR5, and TIM4 in CRC tissues were significantly lower than those in normal tissues, while those of IDO1, TGFβ1, and NOS2 showed no difference (**Supplementary Figure S1B**). However, the data for CCL11 and CCL22 are not available in the HPA database. Our results suggested that the expression levels of the model proteins had similar trends to the expression levels of the corresponding model genes. The patients were divided into the low- and high-risk categories based on the median risk score value generated using the risk formula. More death events were observed in the high-risk group, which suggests that low-risk patients had a better clinical outcome than high-risk patients (**Figure 2B**). As shown by univariate Cox regression analysis in patients with CRC, among the included genes, four (NOS2, HSPA8, HSPA9, and CCL22) were protective factors with HR (hazard ratio) < 1, whereas HSPA1A was the risk factor with HR > 1 (**Figure 2C**). The heat map showed that NOS2, CCL22, CCL28, HSPA8, and HSPA9 expression levels markedly decreased with raised risk scores, while HSPA1A expression significantly increased along with the increased risk scores (**Figure 2D**).

To examine whether clinicopathological features are associated with the risk signature, risk scores were generated for the training set (TCGA) and two independent validation groups (GSE39582, GSE37892). In TCGA cohort, CRC patients with clinical stage IV, CMS4, and advanced TNM stage had higher risk scores (**Figure 2E** and **Supplementary Figure S2**), while age, gender, race, histologic type, and MSI status had no bearing on the risk score (**Supplementary Figure S2**). Likewise, patients with advanced clinical stage and CMS4 presented a significantly higher risk score in GSE39582 and GSE37892 (**Figure 2E**). Both in the training set and in the validating groups, the distribution of patients in terms of alive/dead status, clinical stages, and CMS subtypes showed striking disparities between the low- and high-risk groups (**Figure 2F**).

### Prognostic Significance of the Cancer-Immunity Cycle–Based Signature

**Figure 3:**
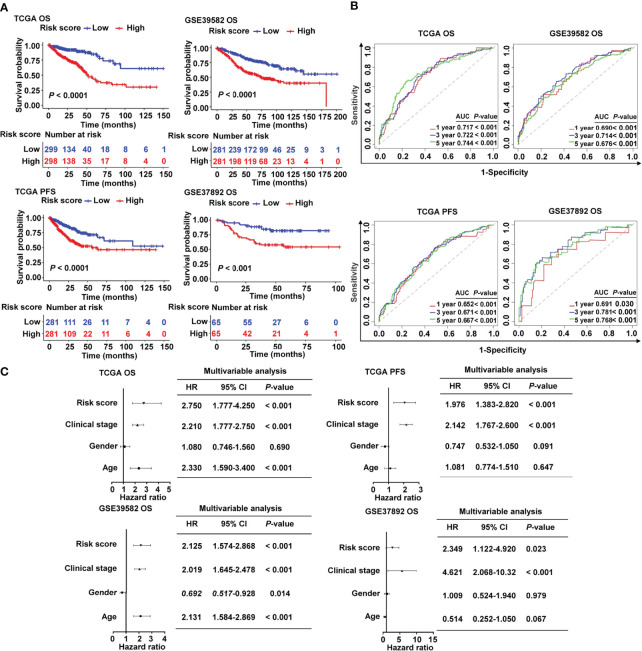
Prognostic value of the cancer-immunity cycle–based signature in CRC patients. **(A)** Kaplan–Meier curves for OS and PFS between the high- and low-risk groups in CRC from TCGA and different GEO data sets. **(B)** ROC curves to evaluate the specificity and sensitivity of one-, three-, and five-year OS and PFS according to the risk score in TCGA and GEO data sets. **(C)** Multivariate Cox analyses of the risk score and clinicopathological parameters for OS and PFS in TCGA and GEO data sets. OS, overall survival; PFS, progression-free survival; HR, hazard ratio; CI, confidence interval.

Patients in the high-risk group had a lower survival rate than those in the low-risk group, according to the Kaplan–Meier (KM) survival curves, which was observed both in TCGA and in two GEO cohorts (**Figure 3A**). Based on the ROC analysis, the risk signature had a potential capability to predict OS in TCGA cohort [AUC of one-year OS = 0.717; AUC of three-year OS = 0.722; AUC of five-year OS = 0.744; **Figure 3B**]. Similarly, the risk signature had relatively high AUC values in the two external validation groups, indicating a good prediction accuracy (**Figure 3B**). ROC curves were used to compare the prediction performance of the risk signature with other clinical parameters. We showed that our risk score model was superior to other clinical parameters for predicting the OS of CRC patients in the TCGA cohort (**Supplementary Figure S3A**) and GSE37892 cohort (**Supplementary Figure S3B**), and it showed the maximum AUC value. The Cox analysis, both univariate and multivariate, was utilized to investigate if age, gender, clinical stage, and risk score of CRC patients were independent prognostic factors (**Supplementary Figure S4** and **Figure 3C**). The forest plot results confirmed that clinical stage and risk score were independent risk factors for predicting OS and PFS (**Figure 3C**), indicating their relevance for predicting the prognosis of patients with CRC. In the 13 candidate genes, we found that five cancer-immunity cycle–related genes were significantly associated with survival outcomes by univariate Cox regression analysis. To further verify the risk model based on 13 cancer-immunity cycle–related genes, another risk model comprising five genes was used to evaluate the prognostic values in CRC by ROC curve analysis. The ROC analysis demonstrated that the AUC values for OS in TCGA cohort were 0.734 and 0.665, in the 13-gene signature risk model and 5-gene signature risk model, respectively (**Supplementary Figure S5A**). Meanwhile, relative to the 5-gene signature risk model, the AUC for OS of the 13-gene signature risk model significantly improved from 0.687 to 0.761 in the GSE37892 cohort (**Supplementary Figure S5B**). These results indicate that the risk model of the 13 cancer-immunity cycle–related genes for CRC is considerably reliable in monitoring survival.

### Establishment and Validation of a Nomogram Combined With Clinical Characteristics

**Figure 4:**
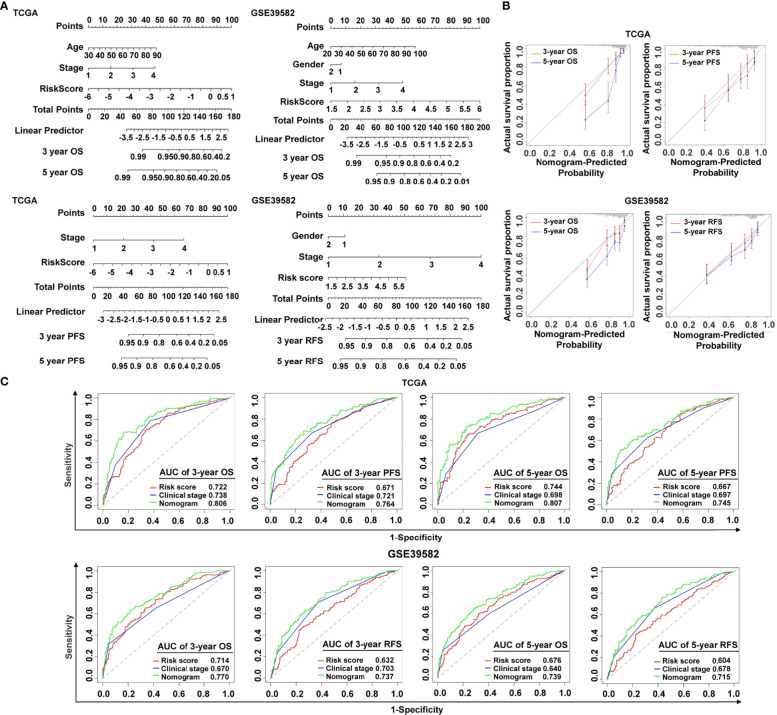
Establishment and verification of the nomogram. **(A)** The predictive nomogram built in combination with the risk signature and clinical characterization predicted three- and five-year survival rates of patients with CRC. OS and PFS prediction for CRC patients in TCGA data set, and OS and RFS prediction for CRC patients in the GSE39582 data set. **(B)** The probabilities of OS, PFS, and RFS at three and five years were assessed by calibration plots of the nomogram in TCGA and GSE39582. **(C)** ROC curves of the nomograms compared with those of other clinical variables with regard to three- and five-year survival in TCGA and GSE39582. OS, overall survival; PFS, progression-free survival; RFS, relapse-free survival.

To make the cancer-immunity cycle–based risk signature more clinically applicable and usable, we established a prognostic nomogram using the risk status and common clinical characteristics, with the goal of developing a quantitative analytic algorithm that can predict individual CRC patients’ expected survival. Each element (age, gender, clinical stage, and risk score) was utilized to calculate the individual sample’s score summary and total score, which can evaluate three- and five-year survival probabilities (**Figure 4A**). To show the consistency between the actual measured prognostic value and the value projected by the nomogram, a calibration curve was utilized. As shown in **Figure 4B**, calibration curves of three-year OS, three-year PFS, and five-year PFS prediction in TCGA cohort were near to optimal performance, indicating the nomogram’s predictive accuracy. The subsequent calibration plots also showed excellent agreement between the predicted probability of three- and five-year OS and the actual OS in the GSE39582 group (**Figure 4B**), with consistent results for three- and five-year RFS prediction observed in the GSE39582 group (**Figure 4B**). ROC analysis was also used to assess the nomogram’s prediction accuracy. The results revealed that, in both the training and the validating sets, the predicted AUC values of the OS, PFS, and RFS nomograms were higher than those of the risk score or the clinical stage (**Figure 4C**), implying that the nomogram outperformed the other predictors when predicting survival of CRC patients. It is also noteworthy that the C-index indicated a stable and robust predictive power of the nomogram in TCGA data set (OS: C-index = 0.783; PFS: C-index = 0.732) and the GSE39582 data set (OS: C-index = 0.714; RFS: C-index = 0.717). These results demonstrated that the nomogram using the cancer-immunity cycle–based signature risk scores was reliable and accurate for predicting the survival of patients with CRC.

### Biological Pathways and Functional Enrichment Analysis of the Cancer-Immunity Cycle–Based Signature

**Figure 5:**
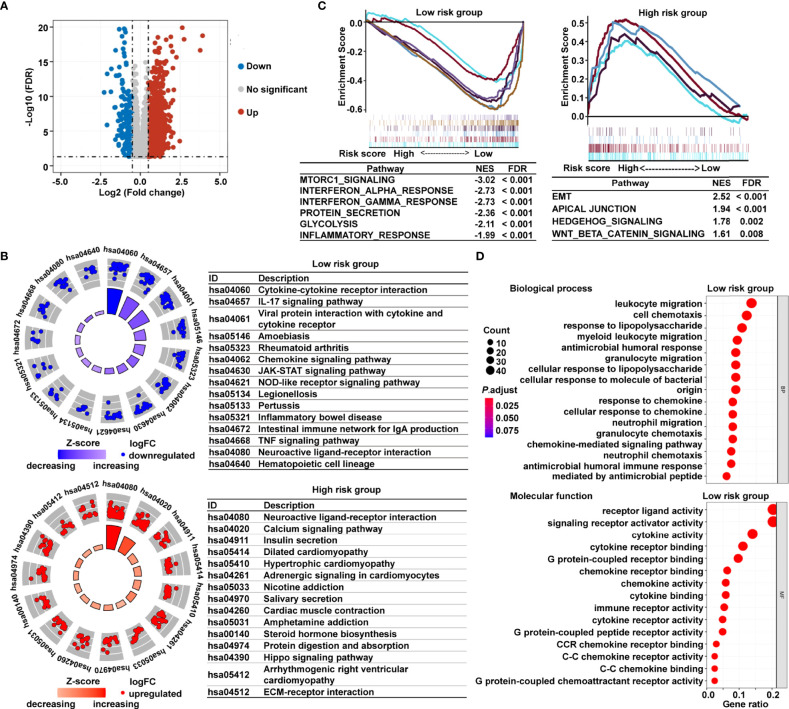
Functional analysis of DEGs based on the cancer-immunity cycle–related signature between low- and high-risk CRC patients. **(A)** Volcano map of DEGs between the low- and high-risk groups from TCGA data set. **(B)** KEGG pathways enriched in the low- and high-risk CRC patients. **(C)** GSEA enrichment plots of the high-risk and low-risk CRC patients. **(D)** The 15 most significantly enriched GO terms in low-risk CRC patients are listed. DEGs, differentially expressed genes.

To investigate the underlying mechanisms that contribute to the different results stratified by the risk signature, we performed KEGG pathway, GSEA, and GO analysis. Volcano plot analysis identified 1794 DEGs between the low- and high-risk subgroups in TCGA cohort (**Figure 5A**). The KEGG pathway enrichment analysis demonstrated that the most significantly altered pathways in the low-risk subgroup were those involving cytokine–cytokine receptor interactions, interleukin 17 (IL-17) signaling pathway, chemokine signaling, and viral protein interactions with cytokines and cytokine receptors (**Figure 5B**). However, patients with high risk scores were mainly converged in tumor-related pathways, for instance, calcium signaling pathway, Hippo signaling pathway, and extracellular matrix (ECM)–receptor interaction (**Figure 5B**). Meanwhile, the GSEA showed that the gene sets involved in interferon γ/α response and inflammatory response were gathered together in low-risk patients; in contrast, signaling pathways promoting tumor progression, including epithelial–mesenchymal transition (EMT), apical junction, Wnt-β-catenin signaling, and Hedgehog signaling, were predominant in high-risk patients (**Figure 5C**). The GO analysis further revealed that many biological functions in low-risk patients primarily correlated with immune-related biological processes and inflammatory reactions (**Figure 5D**). The immunological and inflammatory features of the cancer-immunity cycle–based signature were clearly proven, and the potential mechanism of this risk signature for evaluating the prognosis of patients with CRC was strongly validated using these results.

### Immune Microenvironment and Immune Characterization of the Cancer-Immunity Cycle–Based Signature

**Figure 6:**
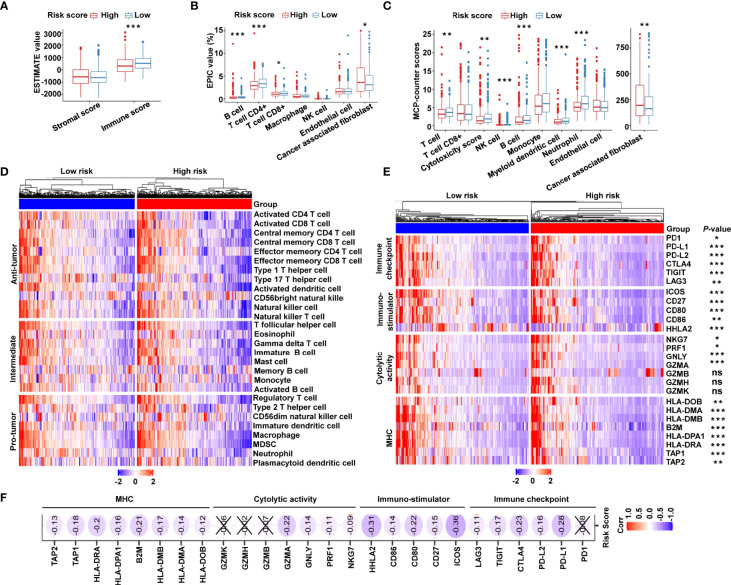
The immune landscape of the cancer-immunity cycle–based signature in CRC. **(A)** Estimation of immune scores and stromal scores between low- and high-risk patients using the ESTIMATE algorithm. **(B)** Estimation of immune cell proportions in low- and high-risk patients using the EPIC algorithm. **(C)** Estimation of proportions of immune cell subsets using the MCP-counter algorithm. **(D)** Heat map displaying the relative proportions of 28 immune cell subsets in low- and high-risk patients estimated using ssGSEA. **(E)** Heat map demonstrating gene expression profiles of immune checkpoint genes, immuno-stimulator signature, cytolytic activity signature, and MHC family genes in low- and high-risk patients. **(F)** Heat map illustrating correlations between risk scores and expression levels of the above corresponding genes. The Spearman correlation coefficient is shown by the number in each oval icon little box. Correlations with a *P* value ≥ 0.05 are marked with a cross. ***P < 0.001; **P < 0.01; *P < 0.05; ns, not significant.

Owing to the tight correlation between the risk signature and immune-related biological pathways, we further investigated the connection between the risk score and tumor-infiltrating immune cells. First, we used ESTIMATE algorithm to quantify the overall infiltrating immune cells based on TCGA cohort. The results showed that the low-risk group exhibited high immune scores, indicating a significantly increased immune cell infiltration in the TME with a low risk score (**Figure 6A**). We further analyzed the specific difference in infiltrating immune cells between the two subgroups. Based on EPIC algorithm, the low-risk subgroup had a higher proportion of B cells, CD4+ T cells, and CD8+ T cells, while cancer-associated fibroblasts (CAFs) were shown to be significantly greater in high-risk patients (**Figure 6B**). In addition, we utilized MCP-counter algorithm. We showed that, compared with the high-risk group, T cells, cytotoxic scores, natural killer (NK) cells, B cells, myeloid dendritic cells, and neutrophils were more abundant, whereas the proportion of CAFs was lower in the low-risk group (**Figure 6C**). To validate the above findings, ssGSEA was conducted to evaluate 28 types of tumor-infiltrating immune cells. The distribution and proportion of different immune cells between the low- and high-risk categories are shown in **Figure 6D**. As expected, antitumor immune cells were considerably more elevated in the low-risk group than in the high-risk group (**Figure 6D**), indicating that the low-risk group had a more active immune response. Furthermore, we investigated the immune microenvironment in the two subgroups using a clustering analysis of immune-related genes. As shown in the heat map (**Figure 6E**), immune checkpoints were markedly upregulated in low-risk patients, and similar trends of cytotoxic molecules and MHC family genes were also observed in low-risk patients. Our analysis illustrated that the risk score was negatively associated with the expression of immune checkpoints, stimulatory immune factors, cytotoxic molecules, and MHC family genes (**Figure 6F**). Our findings revealed substantial variations in intrinsic tumor immunogenicity and anticipated immunotherapy response between the low- and high-risk groups.

### Relationship between the Cancer-Immunity Cycle–Based Signature and Immunotherapy Response

**Figure 7:**
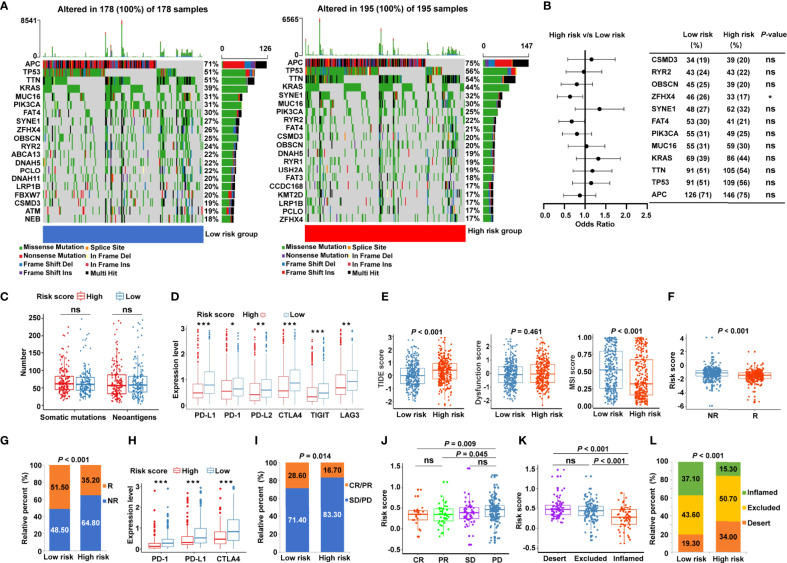
Identification of the cancer-immunity cycle–based signature for predicting immunotherapy response in CRC. **(A)** The waterfall plot of somatic mutation features in low- and high-risk patients using TCGA-COAD data set. **(B)** The forest plot illustrating the differences in the top 10 mutation frequencies of genes in COAD patients of the low- and high-risk groups. **(C)** The number of somatic mutations and neoantigens in low- and high-risk CRC patients from TCGA data set. **(D)** The expression levels of representative immune checkpoint genes in low- and high-risk CRC patients from TCGA cohort. **(E)** The TIDE score, T cell dysfunction score, and MSI score in low- and high-risk CRC patients. **(F)** Comparison of the risk score between distinct immunotherapy responses using the TIDE algorithm in TCGA cohort. R, responder; NR, non-responder. **(G)** The proportions of CRC patients with response to ICIs in the low- and high-risk categories. R, responder; NR, non-responder. **(H)** The expression levels of PD-1, PD-L1, and CTLA4 in low- and high-risk patients using IMvigor210 cohort. **(I)** The proportions of patients with response to PD-L1 blocking immunotherapy in the low- and high-risk categories from IMvigor210 cohort. CR, complete response; PR, partial response; SD, stable disease; PD, progressive disease. **(J)** Distribution of the risk score in distinct responses to anti-PD-L1 treatment in IMvigor210 cohort. **(K)** Differences in the risk score among specific tumor immunophenotype using IMvigor210 cohort. **(L)** The proportion of patients with distinct tumor immunophenotype in the low- and high-risk categories. ***P < 0.001; **P < 0.01; *P < 0.05; ns, not significant.

Given that accumulative evidence has shown that somatic mutations in solid tumors are strongly associated with immunotherapy (29), we investigated the mutational landscape in different risk subgroups. The distribution of somatic mutations was assessed in the low- and high-risk patients from TCGA-COAD (**Figure 7A**) and TCGA-READ (**Supplementary Figure S6A**) cohorts, and each variant’s top 20 commonly altered genes were rated. Our analysis of the mutation data showed that mutation frequencies of the top 10 mutated genes in TCGA-COAD cohort were not substantially different between the low- and high-risk categories, except for the mutation of zinc finger homeobox 4 (ZFHX4) (**Figure 7B**). Similar trends were observed in TCGA-READ cohort, except for the mutation levels of APC regulator of Wnt signaling pathway (APC) (**Supplementary Figure S6B**). Moreover, the risk score showed no significant association with the number of somatic mutations or neoantigens in patients with CRC (**Figure 7C**). To further probe whether the risk signature may play a role in immunotherapy responsiveness, we analyzed the difference in the expression of key immune checkpoints. The results illustrated that immune checkpoints [PD-1, PD-L1, PD-L2, CTLA4, T cell immunoreceptor with Ig and ITIM domains (TIGIT), and LAG3] were highly expressed in the low-risk group (**Figure 7D**). TIDE score, the more accurate predictor for immune checkpoint blockade (ICB) therapies (26), was introduced into our analysis. Interestingly, patients with CRC from the low-risk group had a lower TIDE score (**Figure 7E**) but a higher MSI score (**Figure 7E**) compared with high-risk patients. A greater TIDE score suggests a higher probability of tumor immune escape and lower likelihood of benefitting from anti-PD-1/CTLA4 therapy (26), illustrating that low-risk patients are candidates for ICB therapy. According to the TIDE algorithm, risk scores were significantly elevated in ICB non-responder CRC patients (**Figure 7F**), while the low-risk group had significant therapeutic advantages and clinical response to immunotherapy compared with the high-risk group in TCGA cohort (**Figure 7G**). Furthermore, an immunotherapy cohort, IMvigor210, was also used to investigate whether the risk signature could predict patients’ responses to anti-PD-L1 therapy. Similarly, in the IMvigor210 validation cohort, low-risk patients had greater expression of PD-1, PD-L1, and CTLA4 (**Figure 7H**), and were more sensitive to anti-PD-L1 therapy than high-risk patients (**Figure 7I**). Meanwhile, risk scores were significantly increased in patients with progressive disease (PD) compared with those with complete response (CR) or partial response (PR) (**Figure 7J**). More specifically, we found that the lower risk scores were remarkably associated with inflamed immune phenotype (**Figure 7K**), whereas the proportions of exclusion and desert immune phenotypes were remarkably higher in high-risk patients than in low-risk patients (**Figure 7L**), indicating the difficulty of checkpoint inhibitors to exert antitumor effect in these phenotypes. These results suggested that the cancer-immunity cycle–based signature was able to identify low-risk patients who may benefit from ICB.

### Validation of the Cancer-Immunity Cycle–Based Signature in an Independent Cohort

**Figure 8:**
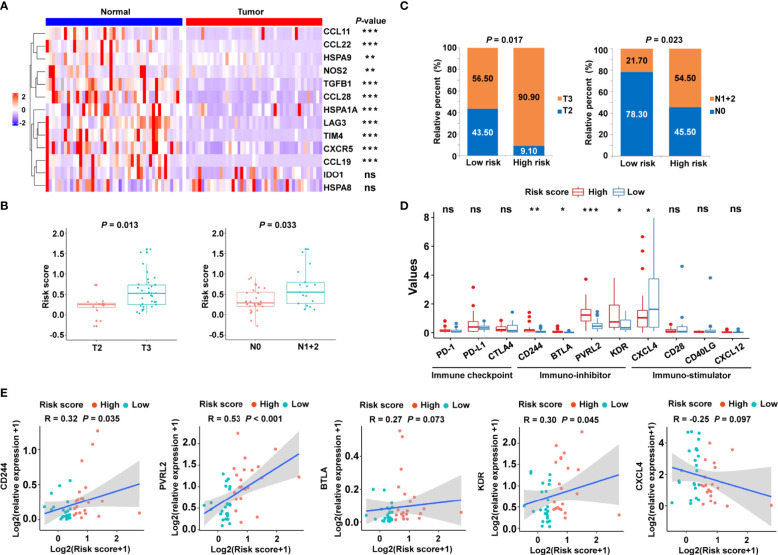
Validation of the cancer-immunity cycle–based signature in an independent CRC cohort. **(A)** Heat map of the gene profiles involved in 13 cancer-immunity cycle–related genes in 45 pairs of CRC tumor tissues and adjacent normal tissues. **(B)** The relationship between clinicopathological characteristics and the risk score defined by the cancer-immunity cycle based-signature in patients with CRC. T stage: the depth of tumor infiltration; N stage: the extent or number of metastasized lymph nodes. **(C)** Histogram showing the ratio of T2 vs. T3, N0 vs. N1+2 between the low- and high-risk groups. **(D)** RT-qPCR assay was performed to examine the relative mRNA levels of immune checkpoint genes (PD-1, PD-L1, and CTLA4), immuno-inhibitor molecules (CD244, BTLA, PVRL2, and KDR), and immuno-stimulator molecules (CXCL4, CD28, CD40LG, and CXCL12) in low- and high-risk patients with CRC. ***P < 0.001; **P < 0.01; *P < 0.05; ns, not significant. **(E)** Correlation between the risk score and the immuno-inhibitor or immuno-stimulator molecules. The Spearman correlation coefficients (R) and corresponding *P* values are shown.

To confirm the clinical significance of the risk signature, RT-qPCR was performed to examine the expression of related genes in 45 pairs of CRC tumor tissues and corresponding normal tissues. The heat map showed the expression levels of 13 cancer-immunity cycle–related genes in frozen tissue samples (**Figure 8A**). Using the risk formula, patients with CRC were divided into two subgroups according to their median risk score. Patients with CRC belonging to T3 or N1+2 showed higher risk scores than those with T2 or N0, respectively (**Figure 8B**). In addition, the risk score significantly correlated with tumor grade and lymph node metastasis (**Figure 8C**). However, the risk score did not significantly correlate with age and gender in the validation cohort (**Supplementary Figure S7**). Interestingly, high-risk patients had higher mRNA levels of immunosuppressive molecules, including CD224, B and T lymphocyte associated (BTLA), poliovirus receptor-related 2 (PVRL2), and kinase insert domain receptor (KDR) (**Figure 8D**). Conversely, immuno-stimulator gene, such as CXCL4, were highly expressed in the low-risk group compared with the high-risk group (**Figure 8D**). In particular, the expression levels of PD-1, PD-L1, and CTLA4 were statistically similar in low- and high-risk patients (**Figure 8D**), which might be related to the limited sample size. In addition, correlation analysis was also performed, and the risk score was found to significantly positively correlate with immune-inhibitor molecules, including CD224, PVRL2 and KDR (**Figure 8E**).

### Analysis of the Correlation Between the Cancer-Immunity Cycle–Based Signature and Drug Sensitivity

**Figure 9:**
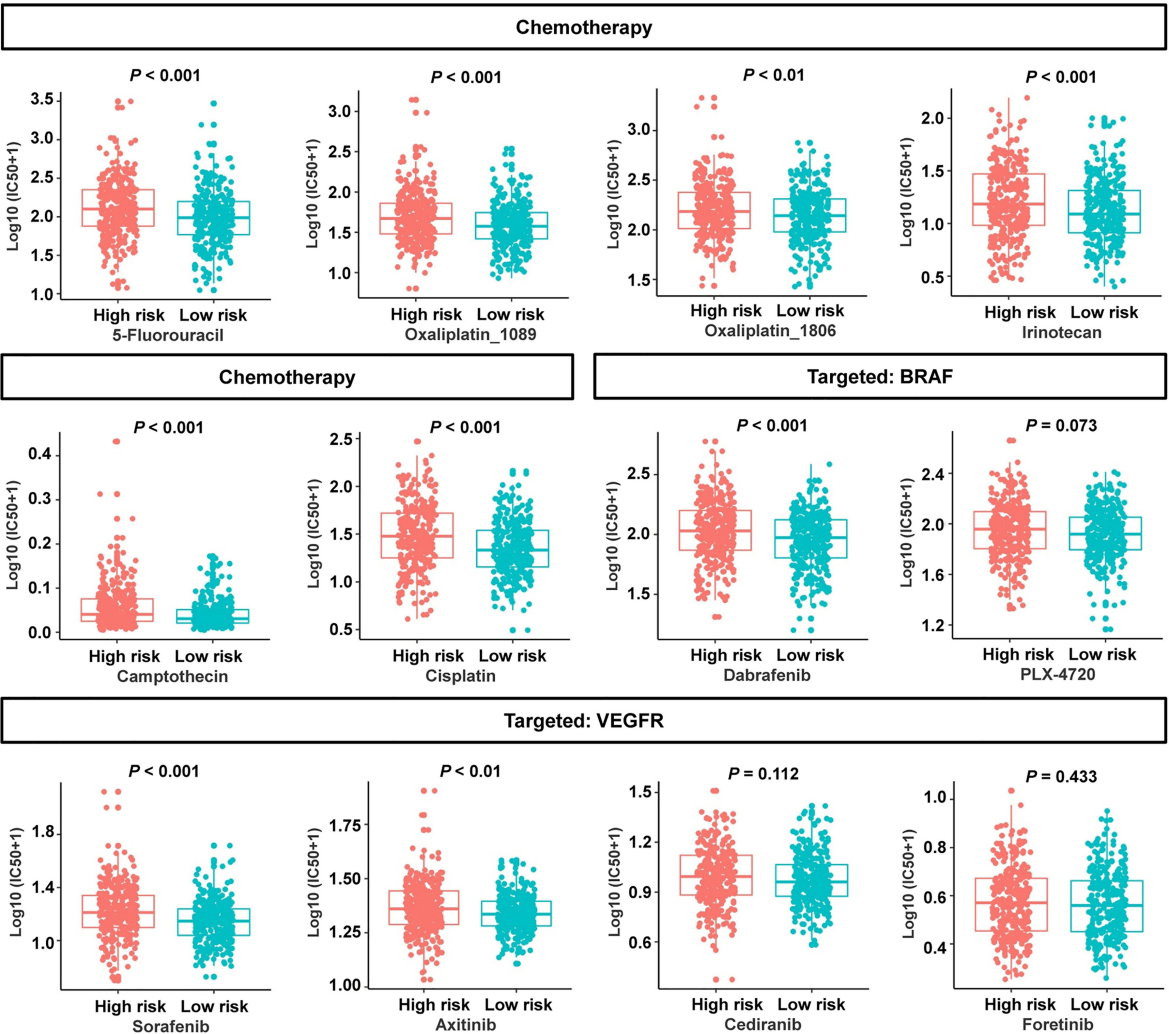
Association between the cancer-immunity cycle–based signature and drug sensitivity, including chemotherapeutics and small molecular drugs targeting BRAF and VEGFR. IC50: half-maximal inhibitory concentration.

To evaluate the risk signature’s usefulness in clinical therapy, we analyzed the chemotherapeutic drug sensitivity in low- and high-risk patients. Interestingly, the patients in the low-risk group exhibited lower half-maximal inhibitory concentration (IC50) values for 5-fluorouracil, oxaliplatin_1089, oxaliplatin_1806, irinotecan, camptothecin, cisplatin, B-Raf proto-oncogene serine/threonine kinase (BRAF) inhibitor dabrafenib, and vascular endothelial growth factor receptor (VEGFR) inhibitor sorafenib or axitinib (**Figure 9**). Three molecular-targeted drugs displayed no statistically significant differences between the two subgroups in terms of the estimated IC50 values (**Figure 9**). In view of these data, the low-risk group might be more sensitive to common chemotherapeutic agents and molecular-targeted drugs. These results suggested that the risk signature can, to a certain extent, predict drug sensitivity in patients with CRC.

## Discussion

PD-L1 expression on tumor and immune cells has been intensively explored as a biomarker for ICI response in a number of different cancers. However, in treatment studies targeting mCRC, PD-L1 expression on tumor or immune cells has not been associated with ICI responses (30–32). Additionally, several other factors such as TMB (33), MSI (34), somatic copy-number alterations (35), T cell signaling (36), human leukocyte antigen (HLA) class I genotype (37), and TGFβ signaling (20) have been shown to correlate with the clinical outcome of ICIs therapy, based on molecular profiling of cancers treated with different immunotherapies. Unlike conventional cancer therapies, ICIs do not directly kill tumor cells; instead, they affect tumor cells through the patient’s immune system or the TME. Therefore, different factors that affect the cancer-immunity cycle and the immunological microenvironment need to be taken into account when developing predictive biomarkers for ICIs in CRC treatment.

In this study, we established a 13-gene signature, composed of five chemokine and chemokine receptor family members (CCL11, CCL19, CCL22, CCL28, CXCR5), three immune checkpoint genes (IDO1, LAG3, TIM4), three HSP70 family members (HSPA1A, HSPA8, HSPA9), and two cytokines (NOS2 and TGFβ1). Chemokines and members of the chemokine receptor family have been shown to be extensively involved in tumor development and have potential value in tumor immunotherapy (38, 39). Our previous study has demonstrated that the combined IDO1 and CD8A classifier was more accurate in predicting clinical outcomes than the known methods for molecular subtyping identification in patients with colon cancer (40). Higher levels of IDO1 have also been detected in hypermutated colorectal cancers, suggesting a link with clinical benefit who receives anti-PD-1, anti-PD-L1, and anti-CTLA4 treatment (41). Additionally, given that CRC is a cancer with high expression of LAG3, targeting LAG3 may be an excellent therapeutic approach to treat such solid tumors (42). Moreover, elevation of TIM4 promotes proliferation and tumor stroma remodeling in CRC, thereby accelerating tumor progression (43). The prognostic significance of distinct HSP70 family members has been reported in colon cancer (44). Recent studies have highlighted the role of the TGFβ pathway activation in inducing immune evasion and primary resistance to ICIs therapy (20, 45, 46), thereby suggesting its evaluation as a predictive biomarker. Although the roles and expression levels of these molecules in numerous cancer types have been reported, the clinical significance of integrating these genes in CRC remains unrevealed. The above studies supported the novel cancer-immunity cycle–related signature as a potentially measurable prognostic indicator in patients with CRC, which may provide a theoretical basis for predicting immunotherapy.

It should be emphasized that we used the median value of the calculated risk score as a cutoff point for distinguishing the low- and high-risk groups in both the training and the validation cohorts. There were several explanations: 1) The median value is a conservative way to avoid the risk of data manipulation and present a comparative objective result. 2) For all cohort analyses, the small number of patients in the low- or high-risk groups would likely affect the prognostic values of the risk signature if optimization of the cutoff was attempted. 3) The universality of the developed model is largely limited by the optimal cutoff value. It is more helpful to objectively evaluate the clinical significance of the risk model according to the median risk score. Thus, other signature-based studies have also divided patients using the median value as the risk score cutoff for predicting survival and immunotherapy efficacy in cancer (47, 48). The prognostic significance of the cancer-immunity cycle–based signature was successfully established in several independent datasets, thereby promoting the exploration of biological mechanisms. Biological pathway and functional enrichment analyses illustrated that immune-related processes and inflammatory activities were significantly converged in low-risk patients, but the special cancer signaling pathways were mainly distributed in high-risk patients. Our findings suggested that immunological heterogeneity between the low- and high-risk groups may be the primary reason for the disparity in clinical outcomes, supporting the risk model’s potential mechanism for predicting CRC prognosis. To discover the connection between tumor-infiltrating immune cells and the risk model, we used various methods to estimate the immune cell infiltration, including ESTIMATE, EPIC, MCP-counter, and ssGSEA algorithm. By integrative analysis, our results revealed that low-risk patients exhibited high immune score and high infiltration by B cells, CD8+ T cells, CD4+ T cells, and NK cells, suggesting that these individuals may be characterized with stronger antitumor immunity activity. Simultaneously, high-risk patients had an immunosuppressive microenvironment with the presence of numerous CAFs. In CRC, infiltration by B cells is an independent predictor of a favorable clinical outcome (49). Meanwhile, a large infiltration of activated CD8+ and CD4+ T cells is linked to favorable survival outcome in patients with colon cancer (50–52). Furthermore, CAFs contribute to an immunosuppressive TME, thereby inducing chemoresistance and worse survival in patients with CRC (53–55). Consistent with previous publications, our study demonstrated the accuracy of our immunological phenotype categorization for different risk scores according to the prognostic value of the risk signature in CRC.

Interestingly, our study highlighted the potential role of the cancer-immunity cycle–based signature in predicting the response to immunotherapy in CRC. The immune-inflamed phenotype is defined as the presence of a large number of immune cell infiltrates in the parenchyma or stroma of a tumor, and it characterizes so-called hot tumors, which have been linked to a better response to ICIs treatment (9, 56). In this study, low-risk patients possessed higher immunological scores and stronger immune activities than high-risk patients, indicating that ICI treatment might provide a greater benefit. Different well-validated immunotherapy biomarkers were employed to further examine the risk signature’s prediction potential. Our results displayed that the expression of immune checkpoint molecules, such as PD-1, PD-L1, and CTLA4, was remarkably increased in low-risk individuals, although somatic mutations and neoantigen levels were not different between low- and high-risk patients. TIDE score is a recently discovered approach for predicting immunotherapy response that is considered to be more accurate than PD-L1 expression or TMB (26). High-risk CRC patients exhibited a higher TIDE score and a lower MSI score when compared to low-risk CRC patients. The greater TIDE score is linked to the greater possibility for immune evasion and the lower chance of immunotherapy benefits (26), which helps to understand why high-risk patients have a poor prognosis. Meanwhile, the TIDE algorithm predicted significantly higher proportion of ICB responders among low-risk patients, showing a significant difference between non-responders and responders. In IMvigor210 cohort, these results were well validated. Namely, low-risk patients had a higher proportion of immune-inflamed phenotype, suggesting that they may have a better response to ICIs. To further validate the results of the bioinformatics analysis, the mRNA expression of the corresponding model genes was measured with RT-qPCR using a clinical CRC cohort. The experimental results showed that the expression levels of CCL11, CCL28, HSPA1A, LAG3, TIM4, CXCR5, and CCL19 in CRC were significantly lower than those in normal tissues but no significant difference was found in the expression of IDO1, which was consistent with TCGA data set. The risk score was calculated for each patient in the validation cohort according to the formula and coefficient obtained from TCGA cohort. Forty-five patients were identified as the low- and the high-risk groups according to the median risk score. We further analyzed the relationship between the risk scores and the clinicopathological features of CRC. These results revealed that a higher risk score was significantly related to a higher tumor stage and metastatic lymph nodes, whereas the risk score was not associated with age or gender. Further validation of CRC samples showed that the risk score positively correlated with the expression of immune-inhibitor molecules but was negatively associated with the expression of immuno-stimulator gene. These findings suggested that high-risk patients were characterized by the aggressive clinical features and tumor immune evasion potential, while low-risk patients were characterized by the immune activation state and were more likely to benefit from ICIs. Thus, the clinical significance of the novel risk signature in the validation cohort was in accordance with that of TCGA cohort. The bioinformatics prediction combined with the experimental validation indicated that our cancer-immunity cycle–based signature might serve as a promising tool for identifying CRC patients suitable for the treatment with ICIs. Additionally, the analysis of drug sensitivity showed significant differences between low- and high-risk patients in response to some chemotherapeutic agents and molecular-targeted drugs. These results illustrated that the risk signature might be helpful in providing guidance for the use of chemotherapy and targeted therapy. As a result, an approach optimizing regimens of a combination of immunotherapy, chemotherapy, and targeted therapy based on the novel cancer-immunity cycle–based signature may be effective for the individualized treatment of patients with CRC.

Despite the promising results, the present study had several limitations. First, although this signature was evaluated and validated in multiple data sets and fresh specimens, a multicenter and large-scale prospective study is needed to confirm our findings. Second, because all of the samples in our investigation were gathered retrospectively, the results may have been impacted by an inherent case selection bias. Some critical clinical data were either incomplete or unavailable for analysis, which might have caused errors. Third, the potential of this signature to predict immunotherapy response was evaluated indirectly because mRNA expression data from CRC patients receiving immunotherapy were not available. In the future, *in vivo* and *in vitro* investigations should be conducted to validate the risk signature.

In conclusion, we comprehensively explored and validated the predictive efficacy of the novel cancer-immunity cycle–based signature on the prognosis of patients with CRC in public cohorts and clinical samples. More importantly, our results suggest that this signature may be a predictor of response to ICIs, which might be helpful in identifying patients who would benefit from antitumor immunotherapy. This may facilitate individual risk stratification and offer innovative perspectives into tailored immunotherapy for patients with CRC.

## Data Availability Statement

The original contributions presented in the study are included in the article/**Supplementary Material**. Further inquiries can be directed to the corresponding author.

## Ethics Statement

The study involving human participants was reviewed and approved by the Research Ethics Committee of The Affiliated Hospital of Qingdao University. The patients/participants provided their written informed consent to participate in this study.

## Author Contributions

Conception and design: MY and YH. Administrative support: MY. Provision of study materials or patients: JZ, WW, MZ, and ZY. Collection and assembly of data: YH, JZ, TL, and WG. Data analysis and interpretation: YH, RZ, SL, and ZZ. Article writing: MY, YH, and RZ. The final manuscript was read and approved by all authors. All authors contributed to the article and approved the submitted version.

## Funding

This work was supported by grants from CAMS Innovation Fund for Medical Sciences (CIFMS) (No. 2021-I2M-1-028) and the Natural Science Foundation of China (NSFC) (No. 81773750).

## Conflict of Interest

The authors declare that the research was conducted in the absence of any commercial or financial relationships that could be construed as a potential conflict of interest.

## Publisher’s Note

All claims expressed in this article are solely those of the authors and do not necessarily represent those of their affiliated organizations, or those of the publisher, the editors and the reviewers. Any product that may be evaluated in this article, or claim that may be made by its manufacturer, is not guaranteed or endorsed by the publisher.
