# Supplementary material for: Comprehensive Analysis of a Cancer-Immunity Cycle–Based Signature for Predicting Prognosis and Immunotherapy Response in Patients With Colorectal Cancer
Source: Front Immunol. 2022 May 31;13:892512. doi: 10.3389/fimmu.2022.892512 (PMC9193226; doi:10.3389/fimmu.2022.892512)
Supplement: Supplementary file 1 [file DataSheet_1.docx]

**SUPPLEMENTARY MATERIALS AND METHODS**

**Signature Construction**

We performed LASSO regression and multivariate Cox regression analysis to identify significant prognostic cancer-immunity cycle–related genes and create a risk score model. Followed by LASSO regression analysis, 10-fold cross-validation was used to build the risk model. With an optimal λ value of 0.032, 13 cancer-immunity cycle–associated genes remained according to the minimum partial likelihood deviance. We then performed multivariate Cox regression analysis on the 13 cancer-immunity cycle–associated genes, including five chemokine and chemokine receptor family members (CCL11, CCL19, CCL22, CCL28, CXCR5), three immune checkpoint genes (IDO1, LAG3, TIM4), three HSP70 family members (HSPA1A, HSPA8, HSPA9), and two cytokines (NOS2 and TGFβ1). The signature risk score was constructed as follows: Risk score = (–0.0471* expression of CCL11) + (0.0139* expression of CCL19) + (–0.2407* expression of CCL22) + (–0.0168* expression of CCL28) + (0.9842* expression of CXCR5) + (0.0037* expression of IDO1) + (0.0617* expression of LAG3) + (0.3028* expression of TIM4) + (0.0073* expression of HSPA1A) + (–0.0005* expression of HSPA8) + (–0.0097* expression of HSPA9) + (–0.0144* expression of NOS2) + (0.0125* expression of TGFβ1).

Univariate Cox regression was conducted to analyze the 13 genes associated with prognosis from the TCGA database, showing that five cancer-immunity cycle–related genes were significantly associated with the survival. Subsequently, multivariate Cox analysis was performed to construct the risk model of the 5-related genes using the survival R package. The following formula was used to calculate the risk score for each patient: Risk score = (–0.1246* expression of CCL22) + (0.0075* expression of HSPA1A) + (–0.0008* expression of HSPA8) + (–0.0096* expression of HSPA9) + (–0.0142* expression of NOS2). ROC analysis was conducted to determine the prognostic value of the risk model.

**SUPPLEMENTARY FIGURES**

**
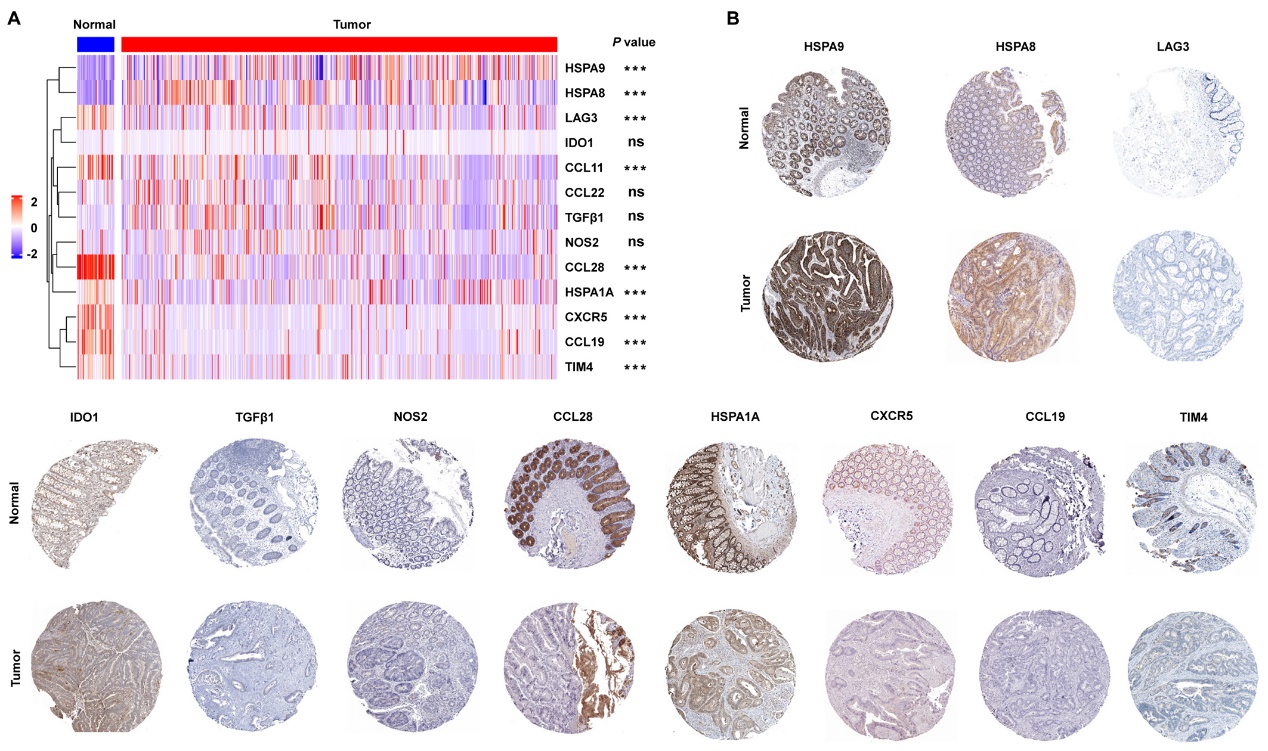
**

**Supplementary Figure S1** Differences in the expression of 13 cancer-immunity cycle–related genes in normal tissues and CRC tissues. **(A)** Gene expression heat map in normal tissue and CRC tumor samples from TCGA data set. **(B)** Protein expression map of cancer-immunity cycle–related genes in the HPA database. ****P* < 0.001; ns: not significant.

**
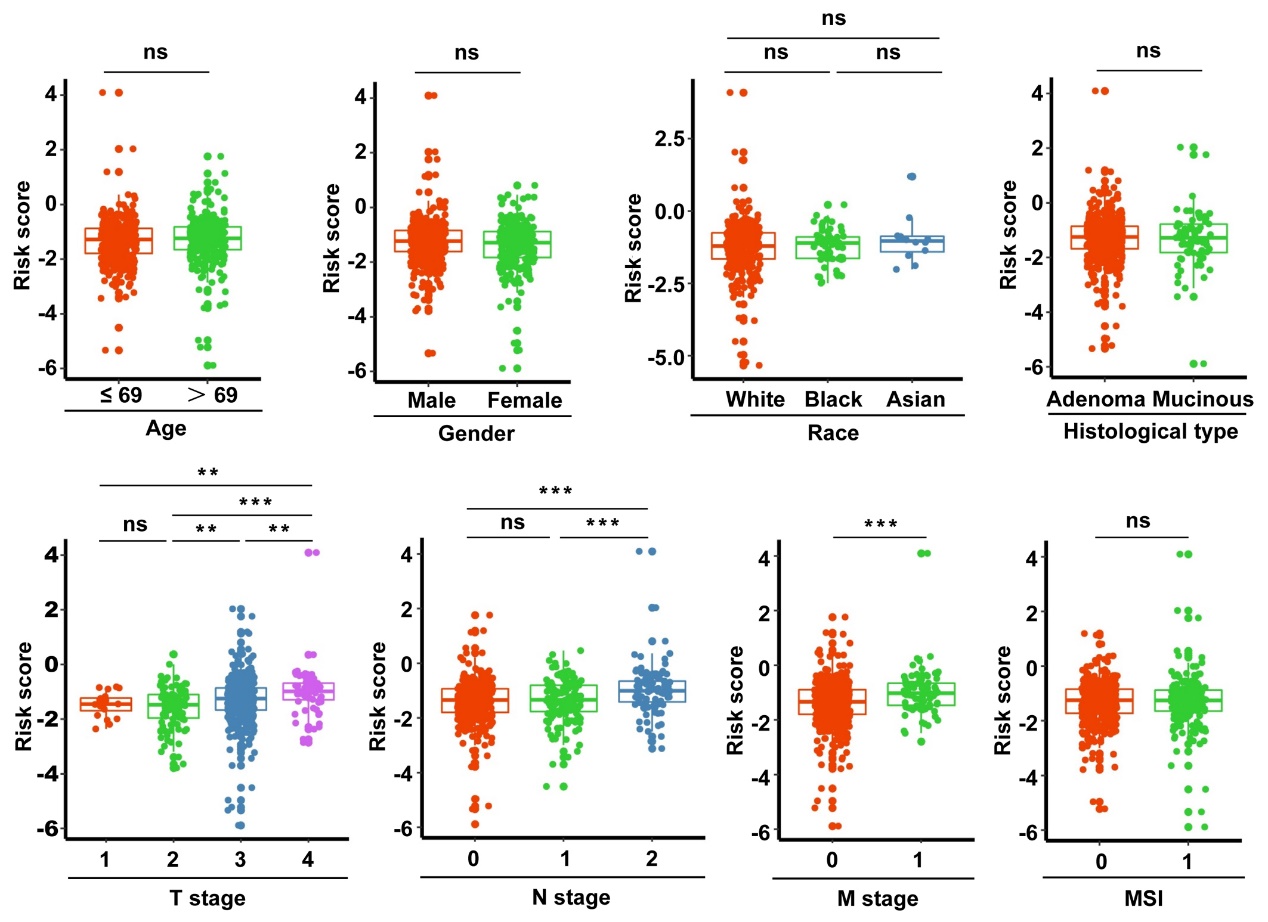
**

**Supplementary Figure S2** The association between the risk score and clinicopathological parameters in TCGA-CRC data set. T stage: the depth of tumor infiltration; N stage: the extent or number of metastatic lymph nodes; M stage: the extent of distant metastasis; MSI: microsatellite instability. ****P* < 0.001; ***P* < 0.01; ns: not significant.


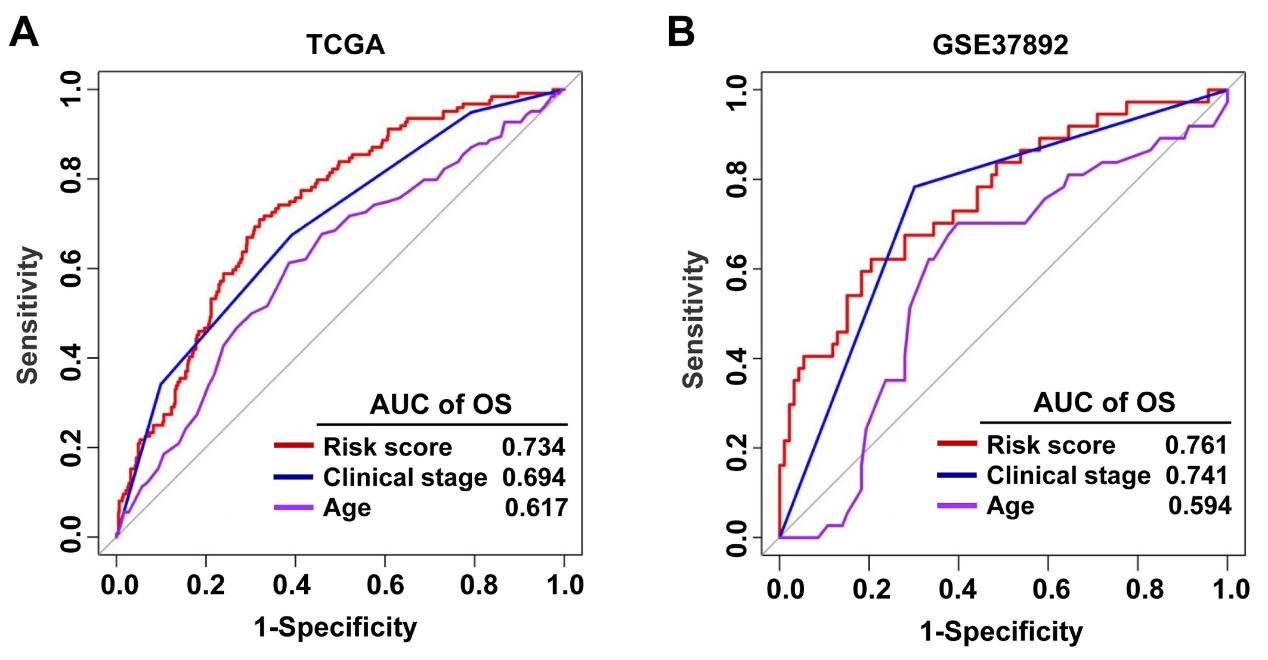


**Supplementary Figure S3** ROC curves for predicting OS with regard to the risk score model and other clinical characteristics in TCGA **(A)** cohort and GSE37892 **(B)** cohort. OS: overall survival.

**
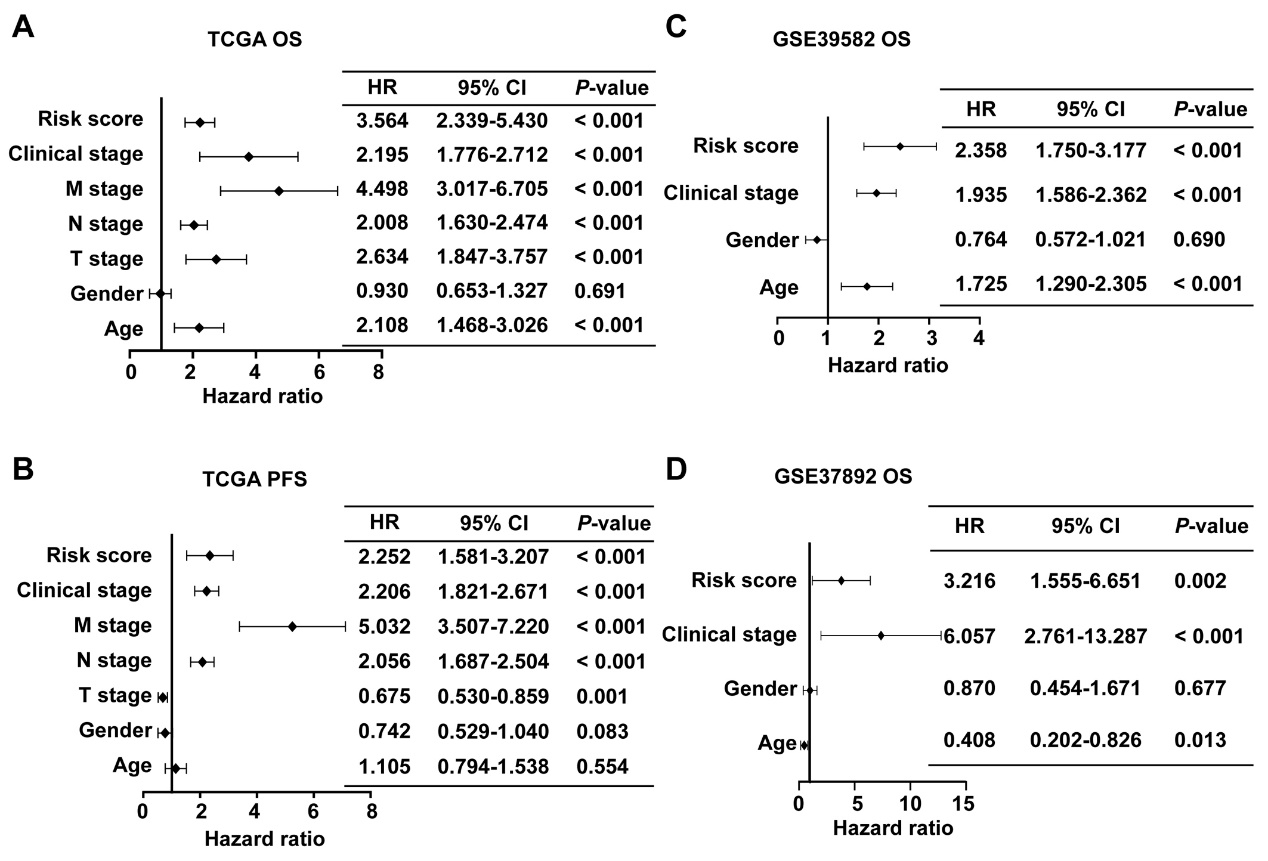
**

**Supplementary Figure S4** Univariate Cox analysis to assess the prognostic value of the cancer-immunity cycle–based signature in CRC patients. **(A)** Overall survival (OS) for the TGGA data set. **(B)** Progression-free survival (PFS) for the TGGA data set. **(C)** OS for the GSE39582 data set. **(D)** OS for the GSE37892 data set. HR: hazard ratio; CI: confidence interval.

**
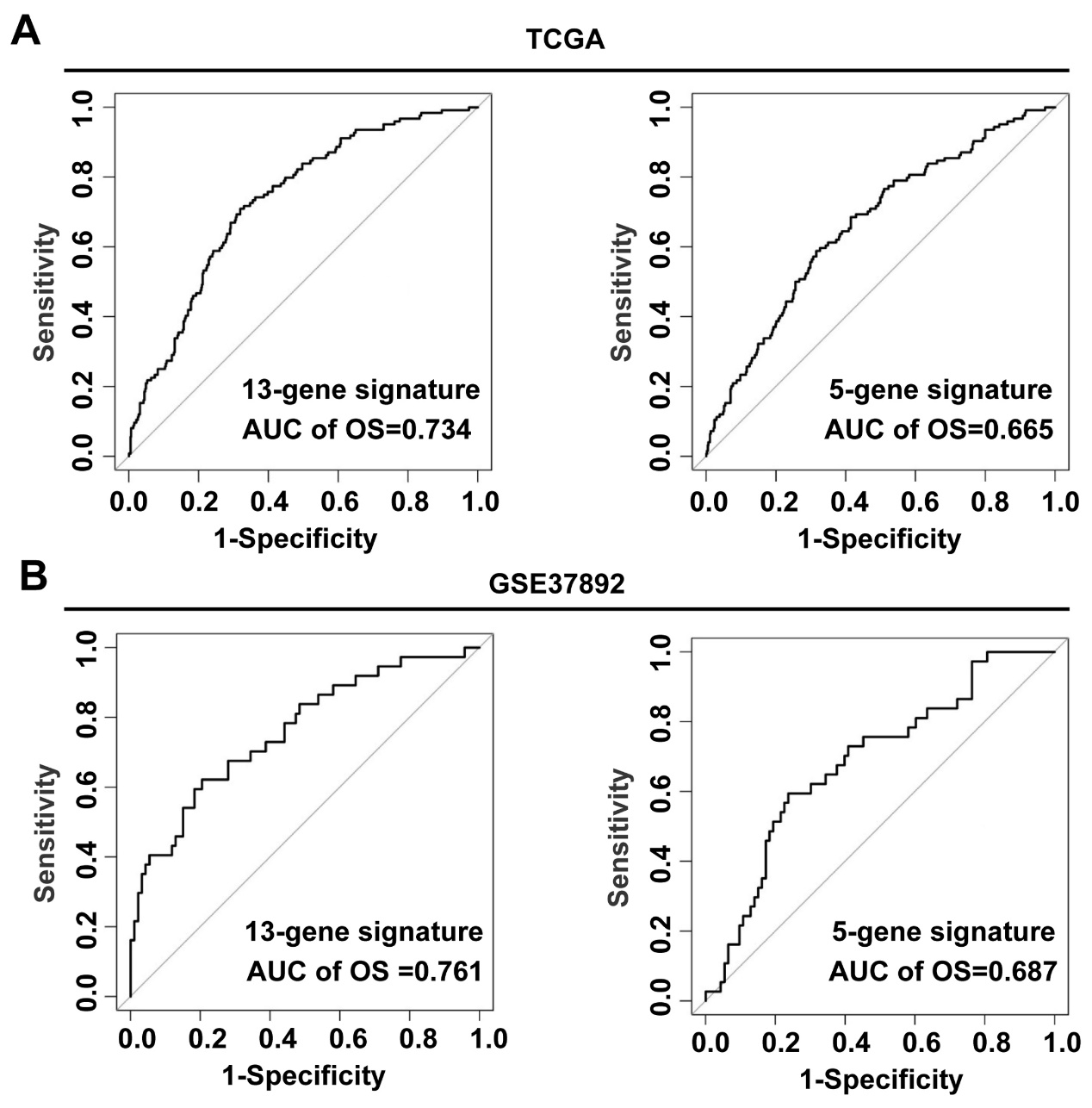
**

**Supplementary Figure S5** The comparison of the ROC curve with the 5-gene signature highlights the superiority of the 13-gene signature risk model in TCGA **(A)** and GSE37892 **(B)** data sets. OS: overall survival.

**
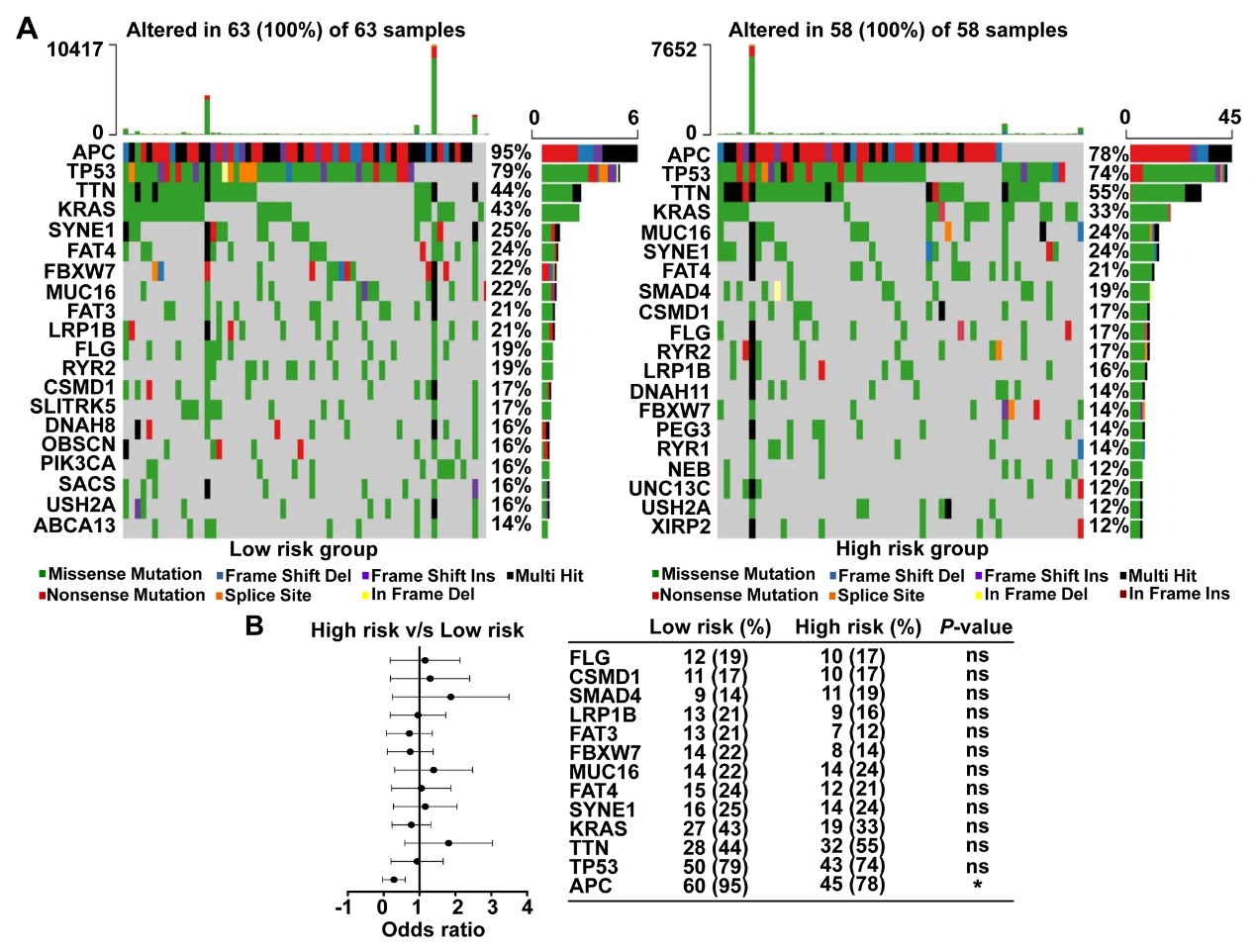
**

**Supplementary Figure S6** Characterization of somatic mutational landscape in patients with rectum adenocarcinoma (READ). **(A)** The waterfall plot of somatic mutation features in low- and high-risk patients using TCGA-READ data set. **(B)** The forest plot illustrates the differences in the top 10 mutation frequencies of genes in READ patients of the low- and high-risk groups.

**
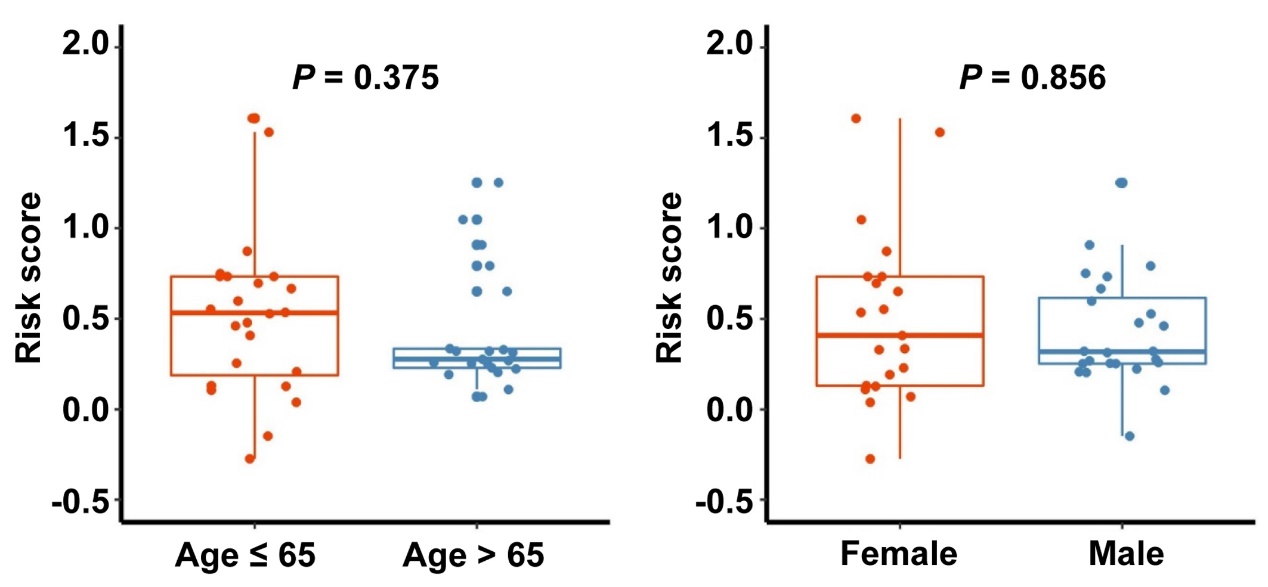
**

**Supplementary Figure S7** The relationship of the risk score with age and gender in CRC patients using the validation cohort.

**Supplementary Table S1** Clinical characteristics of the enrolled patients with CRC.

| Sample_ID | Gender | Age | Size (cm) | Differentiation | Diagnosis | Histologic | T | N | M |
| --- | --- | --- | --- | --- | --- | --- | --- | --- | --- |
| No.1 | Male | 51 | 6.0×5.0 | moderate | right-sided colon cancer | Adenocarcinoma | 2 | 0 | Mx |
| No.2 | Female | 65 | 7.5×4.0 | moderate | right-sided colon cancer | Adenocarcinoma | 3 | 2 | Mx |
| No.3 | Female | 64 | 5.0×5.0 | moderate | colon cancer | Adenocarcinoma | 3 | 1 | Mx |
| No.4 | Female | 74 | 3.8×2.8 | moderate | sigmoid colon cancer | Adenocarcinoma | 2 | 1 | Mx |
| No.5 | Male | 61 | 6.0×5.5 | moderate | colon cancer | Adenocarcinoma | 3 | 2 | Mx |
| No.6 | Female | 74 | 5.0×4.5 | moderate | right-sided colon cancer | Adenocarcinoma | 3 | 0 | Mx |
| No.7 | Male | 81 | 5.5×5.0 | moderate | colon cancer | Adenocarcinoma | 3 | 2 | Mx |
| No.8 | Male | 66 | 2.0×2.0 | moderate | sigmoid colon cancer | Adenocarcinoma | 2 | 1 | Mx |
| No.9 | Male | 82 | 6.5×3.5 | moderate | right-sided colon cancer | Adenocarcinoma | 3 | 0 | Mx |
| No.10 | Female | 45 | 9.5×6.5 | moderate | sigmoid colon cancer | Mucinous Adenocarcinoma | 3 | 1 | Mx |
| No.11 | Male | 62 | 10.0×3.0 | moderate | colon cancer | Adenocarcinoma | 3 | 0 | Mx |
| No.12 | Female | 53 | 6.0×5.5 | moderate | colon cancer | Adenocarcinoma | 2 | 0 | Mx |
| No.13 | Female | 81 | 4.5×4.0 | moderate | right-sided colon cancer | Adenocarcinoma | 3 | 0 | Mx |
| No.14 | Male | 67 | 5.0×3.0 | moderate | right-sided colon cancer | Adenocarcinoma | 3 | 1 | Mx |
| No.15 | Female | 58 | 4.0×2.0 | mid-low | sigmoid colon cancer | Adenocarcinoma | 3 | 1 | Mx |
| No.16 | Male | 60 | 3.0×2.5 | moderate | colon cancer | Adenocarcinoma | 3 | 0 | Mx |
| No.17 | Male | 71 | 5.5×3.5 | moderate | right-sided colon cancer | Adenocarcinoma | 3 | 0 | Mx |
| No.18 | Female | 71 | 7.0×4.0 | moderate | colon cancer | Adenocarcinoma | 3 | 0 | Mx |
| No.19 | Female | 79 | 8.0×7.0 | mid-low | right-sided colon cancer | Adenocarcinoma | 3 | 2 | Mx |
| No.20 | Female | 39 | 5.5×3.0 | moderate | sigmoid colon cancer | Adenocarcinoma | 3 | 0 | Mx |
| No.21 | Female | 65 | 3.0×1.5 | moderate | sigmoid colon cancer | Adenocarcinoma | 3 | 0 | Mx |
| No.22 | Male | 61 | 7.0×5.0 | moderate | right-sided colon cancer | Adenocarcinoma | 3 | 0 | Mx |
| No.23 | Female | 43 | 6.5×5.5 | moderate | sigmoid colon cancer | Adenocarcinoma | 3 | 1 | Mx |
| No.24 | Male | 64 | 3.5×3.0 | moderate | rectum cancer | Adenocarcinoma | 2 | 0 | Mx |
| No.25 | Male | 55 | 1.5×1.5 | moderate | rectum cancer | Adenocarcinoma | 3 | 0 | Mx |
| No.26 | Female | 59 | 5.5×4.0 | moderate | rectum cancer | Adenocarcinoma | 3 | 0 | Mx |
| No.27 | Male | 77 | 7.0×5.5 | moderate | rectum cancer | Mucinous Adenocarcinoma | 2 | 0 | Mx |
| No.28 | Male | 66 | 5.5×4.5 | moderate | rectum cancer | Adenocarcinoma | 3 | 0 | Mx |
| No.29 | Male | 66 | 3.0×3.0 | moderate | rectum cancer | Adenocarcinoma | 2 | 0 | Mx |
| No.30 | Male | 72 | 5.0x4.0 | moderate | rectum cancer | Adenocarcinoma | 3 | 1 | Mx |
| No.31 | Female | 48 | 3.0×2.0 | moderate | rectum cancer | Adenocarcinoma | 2 | 0 | Mx |
| No.32 | Male | 43 | 4.5×3.0 | moderate | rectum cancer | Adenocarcinoma | 2 | 0 | Mx |
| No.33 | Female | 73 | 5.5×4.5 | moderate | rectum cancer | Mucinous Adenocarcinoma | 3 | 1 | Mx |
| No.34 | Female | 76 | 4.0×4.0 | high | rectum cancer | Adenocarcinoma | 2 | 0 | Mx |
| No.35 | Male | 72 | 5.0×3.5 | moderate | rectum cancer | Adenocarcinoma | 3 | 0 | Mx |
| No.36 | Male | 70 | 5.0×5.0 | moderate | rectum cancer | Adenocarcinoma | 3 | 0 | Mx |
| No.37 | Female | 67 | 1.5×1.3 | moderate | rectum cancer | Adenocarcinoma | 2 | 0 | Mx |
| No.38 | Male | 64 | 3.0×3.0 | moderate | rectum cancer | Adenocarcinoma | 3 | 0 | Mx |
| No.39 | Male | 62 | 4.0×4.0 | moderate | rectum cancer | Adenocarcinoma | 3 | 2 | Mx |
| No.40 | Female | 48 | 4.5×4.0 | low | rectum cancer | Adenocarcinoma | 3 | 1 | Mx |
| No.41 | Male | 53 | 4.0×3.0 | moderate | rectum cancer | Adenocarcinoma | 3 | 2 | Mx |
| No.42 | Female | 55 | 3.0×3.0 | moderate | rectum cancer | Adenocarcinoma | 3 | 1 | Mx |
| No.43 | Male | 75 | 4.5×4.5 | moderate | rectum cancer | Adenocarcinoma | 2 | 0 | Mx |
| No.44 | Male | 75 | 5.5×4.0 | high | rectum cancer | Adenocarcinoma | 3 | 0 | Mx |
| No.45 | Female | 53 | 4.5×3.0 | moderate | rectum cancer | Adenocarcinoma | 3 | 0 | Mx |

**Supplementary Table S2** Primers used for RT-qPCR.

| Name of gene | Sequence (5’🡪3’) |
| --- | --- |
| GAPDH F | CTGACTTCAACAGCGACACC |
| GAPDH R | TGAGCTTGACAAAGTGGTCGT |
| HSPA1A F | GGAGCTTCAAGACTTTGCATT |
| HSPA1A R | CAACATTGCAAACACAGGAAATTGA |
| CCL19 F | GACCAGAAGGAAGGACCAGG |
| CCL19 R | TAGCATTGCAATCTGGGGGT |
| CXCR5 F | AACAGTGGGTGGCCATGTAG |
| CXCR5 R | TCTTCAAGTTATTGCTCCCTCCA |
| TIMD4 F | GAAACTGTCCTCCCCAGTGA |
| TIMD4 R | CCAGGCTGAGGAGAAGACAC |
| IDO1 F | TACCCATTGTAACAGAGCCACA |
| IDO1 R | ATGCACAGGTATTTTGAGGTCTT |
| LAG3 F | TGACTGGAGACAATGGCGAC |
| LAG3 R | AGAGAGCTCCACACAAAGCG |
| TGFB1 F | AAGGTGAGGAAACAAGCCCA |
| TGFB1 R | ATCCCCCACTAAAGCAGGTTC |
| CCL11 F | ACCCCTTCAGCGACTAGAGA |
| CCL11 R | CCACTTCTTCTTGGGGTCGG |
| CCL22 F | GAGGCCATTTCACTCCCTGA |
| CCL22 R | CACTTTCAACCTGAGCCCCA |
| CCL28 F | GCACATCAGGGGAAACACGA |
| CCL28 R | AACCAATCATGGCCAAGTCC |
| NOS2 F | GCACCCTGGATTGATCGGAG |
| NOS2 R | TGCACTCAGCAGCAAGTTCC |
| HSPA8 F | TCGTAGCAAATTCTGTGGCAGT |
| HSPA8 R | CTTCCCCTGTGCATATGTTCCA |
| HSPA9 F | ACAGTGGTGTCCTAGTCCGA |
| HSPA9 R | GCTCTGCTTTGATGTGAGTGG |
| PD-1 F | CCCAAGGCGCAGATCAA |
| PD-1 R | GCACTTCTGCCCTTCTCTCTGT |
| CTLA4 F | ACGGGACTCTACATCTGCAAGG |
| CTLA4 R | GGAGGAAGTCAGAATCTGGGCA |
| CD274 F | GGAAATTCCGGCAGTGTACC |
| CD274 R | TGACAGCTGGTGGCATTCAA |
| BTLA_F | ACTGTTTGCCAAAATCACAAATCA |
| BTLA_R | TGCATGTATGTCTCTGACACCA |
| CD244_F | AGGAAAGCGACAAGGGTGAA |
| CD244_R | AAGAACCAGGCTTTGTGCAG |
| KDR_F | ACTATAAGACATGCTATGGCACA |
| KDR_R | TGCTGAAAGCATTATGACCTTTGT |
| PVRL2_F | TCAGACAGGGTGAGCCTCATA |
| PVRL2_R | CTATTCCACTGGGCAAGGGT |
| CXCL4_F | TGAAGAATGGAAGGAAAATTTGC |
| CXCL4_R | CAAATGCACACACGT- AGGCAGCT |
| CD28_F | GAGAAGAGCAATGGAACCATTATC |
| CD28_R | TAGCAAGCCAGGACTCCACCAA |
| CD40LG_F | CAGGCCGTTGCTAGTCAGTT |
| CD40LG_R | GTAATGAGGAGTGGGCAGGC |
| CXCL12_F | TGCAGCTTTTCAATGTTAGCC |
| CXCL12_R | CCCACATACAGTAGGACGTTT |

*Note.* F indicates forward; R indicates reverse.
